# A pilot study exploring novel contexts for out-of-office blood pressure measurement

**DOI:** 10.3389/fcvm.2024.1351746

**Published:** 2024-02-23

**Authors:** Francis Allinson, Nolan Mejia, Lauren Ariniello, Giorgio Quer, Evan D. Muse

**Affiliations:** ^1^ Division of Cardiovascular Disease, Scripps Clinic, La Jolla, CA, United States; ^2^ Scripps Research Translational Institute, La Jolla, CA, United States

**Keywords:** blood pressure, hypertension, digital medicine, personalized medicine, prevention

## Abstract

**Introduction:**

Out-of-office blood pressure (BP) monitoring is increasingly valuable in the diagnosis and management of hypertension. With advances in wearable BP technologies, the ability to gain insight into BP outside of traditional centers of care has expanded greatly.

**Methods:**

Here we explore the usability of a novel, wrist-worn BP cuff monitor for out-of-office data collection with participants following digital cues rather than in-person instruction. Transmitted measurements were used to evaluate BP variation with the time of day and day of week, BP variation with mood, and orthostatic measurements.

**Results:**

Fifty participants, with a mean age of 44.5 years, were enrolled and received the BP monitor. 82% of the participants transmitted data via the smartphone application, and the median wear time of the device during the 4-week study was 11 days (IQR 8-17).

**Discussion:**

This prospective digital pilot study illustrates the usability of wearable oscillometric BP technology combined with digital cues via a smartphone application to obtain complex out-of-office BP measurements, including orthostatic vital signs and BP associated with emotion. 25 out of 32 participants who attempted orthostatic vital signs based on in-app instruction were able to do so correctly, while 24 participants transmitted BP readings associated with emotion, with a significant difference in BP noted between calm and stressed emotional states.

## Introduction

Innovations in mobile technology and connectivity are redefining current medical practices by expanding monitoring and analytical capabilities at home. Indeed, the coronavirus disease of 2019 (COVID-19) pandemic has further accelerated the focus on remote and virtual care at an unprecedented rate (1). Hypertension (HTN) is the most common modifiable risk factor for cardiovascular disease and death worldwide (2). Home BP measurements are recommended by all major medical organizations to reduce bias, namely white coat HTN and masked HTN, and improve the screening and management of HTN (3). In a recent study of nearly 60,000 patients followed for close to 10 years, 24-h ambulatory BP and specifically night-time BP was far more predictive of all cause death and cardiovascular related death when compared to clinic based BP (4). Home measurement devices have not traditionally been designed to be worn all day. Innovation in this space has led to the development of wearable watch-type BP monitoring (WBPM) devices that are worn like a wristwatch. These devices are to be worn throughout the day and can record BP in new contexts (5). They have been verified against traditional arm cuff BP machines and have the potential to be far more convenient than bulkier devices used in the outpatient setting (6–8). With the ability to transmit multiple BP readings per day, these monitors can provide new insights on outpatient BP trends (9).

Many BP devices sync with smartphone applications to provide an interactive platform to review BP trends and access digital cues (10). These advances could aid with complex BP measurements previously confined to the office such as orthostatic BP measurements. Orthostatic hypotension is associated with increased mortality and adverse cardiovascular outcomes, however it is potentially under recognized when only measured in the clinic setting (11). While prior studies have shown that orthostatic BP can be monitored at home, they have relied on in-person instruction for correct measurement technique (12, 13). Emotional stress is increasingly common in modern society and it has been shown to lead to the development and progression of hypertension by various mechanisms (14). WBPM devices have reliably quantified BP changes due to emotion (15).

Our study was conducted in a decentralized fashion, in that data was collected remotely rather than in person at a clinical research facility (16). The aim of our study was to understand the usability of a WBPM devices synced with a smartphone application for routine BP monitoring during day-to-day life and for more complex BP measurements based on cues from a smartphone application rather than in-person instruction including orthostatic BP and BP associated with emotion.

## Methods

### Participants

The institutional review board (IRB) at the Scripps (La Jolla, CA) approved this study and it has been registered at clinicaltrials.gov (NCT03661112). Participants were eligible to join the study if they were a program partner of the All of Us Research Program at the Scripps Research Translational Institute. After all the interested and eligible participants completed multiple screening questions, 50 participants were fully enrolled based on age, gender, and organization affiliation from across the United States and Puerto Rico. All participants provided informed consent to take part in the trial.

### Blood pressure monitoring

After enrolling, participants were mailed a HeartVue (Omron Inc) device, which measures BP based on oscillometric pulses, a charger, and instructions on how to download and use the study application on their smartphone or tablet. Participants were asked to complete the following tasks: wear the device at least two days per week for a total of four weeks, record at least two measurements per week associated with either calm or stressed, and take two orthostatic measurements during the course of the entire trial. BP measurements were logged directly into the study application using Bluetooth connection to the participant's smartphone and securely transmitted to servers via the Connect App interface. Participants were able to flag selected BP measurements as being related to a calm or stressed emotional state or orthostatic testing. A correct orthostatic BP was defined as less than or equal to 3 min between each of 4 consecutive measurements (3 sitting and 1 standing). When analyzing BP variation with time of day, the day was divided into 3 segments, midnight to 8 AM, 8AM to 4 PM and 4 PM to midnight. After using the device for four weeks, participants were asked to return the device and answer a post-study questionnaire (Supplementary Information).

### Statistical analysis

Demographic characteristics of the participants were based on survey responses and analyzed using univariate descriptive statistics (i.e., proportions and means and standard deviations). Univariate descriptive statistics were similarly used to describe average daily BP variation, BP associated with emotion and BP associated with time of the day. To ensure that each individual's BP was weighted equally when calculating daily BP variation, we first averaged the daily BP for each individual who transmitted a minimum of 10 total BP recordings throughout the duration of the study. If participants did not have a BP recording associated with a specific day of the week, then their total average BP (excluding BP measurements associated with emotion and orthostatic measurements) was used for that specific day rather than leaving that day blank. We then took the average of each Individual's average daily BP to come up with the daily systolic and diastolic mean. An ordinary one-way ANOVA using multiple comparisons was used to test for significance of the differences between each pair of average day BP considered, and a similar model was used to test for difference in BP for different times of the day. Average BP associated with emotional state per individual was calculated using a participant's total number of BP readings associated with either calm or stressed emotional state over the 4-week study. An unpaired *T* test was used to calculate significance between stressed and calm emotional states. All statistical analyses were conducted using GraphPad Prism 8 for MacOS.

## Results

**Figure 1:**
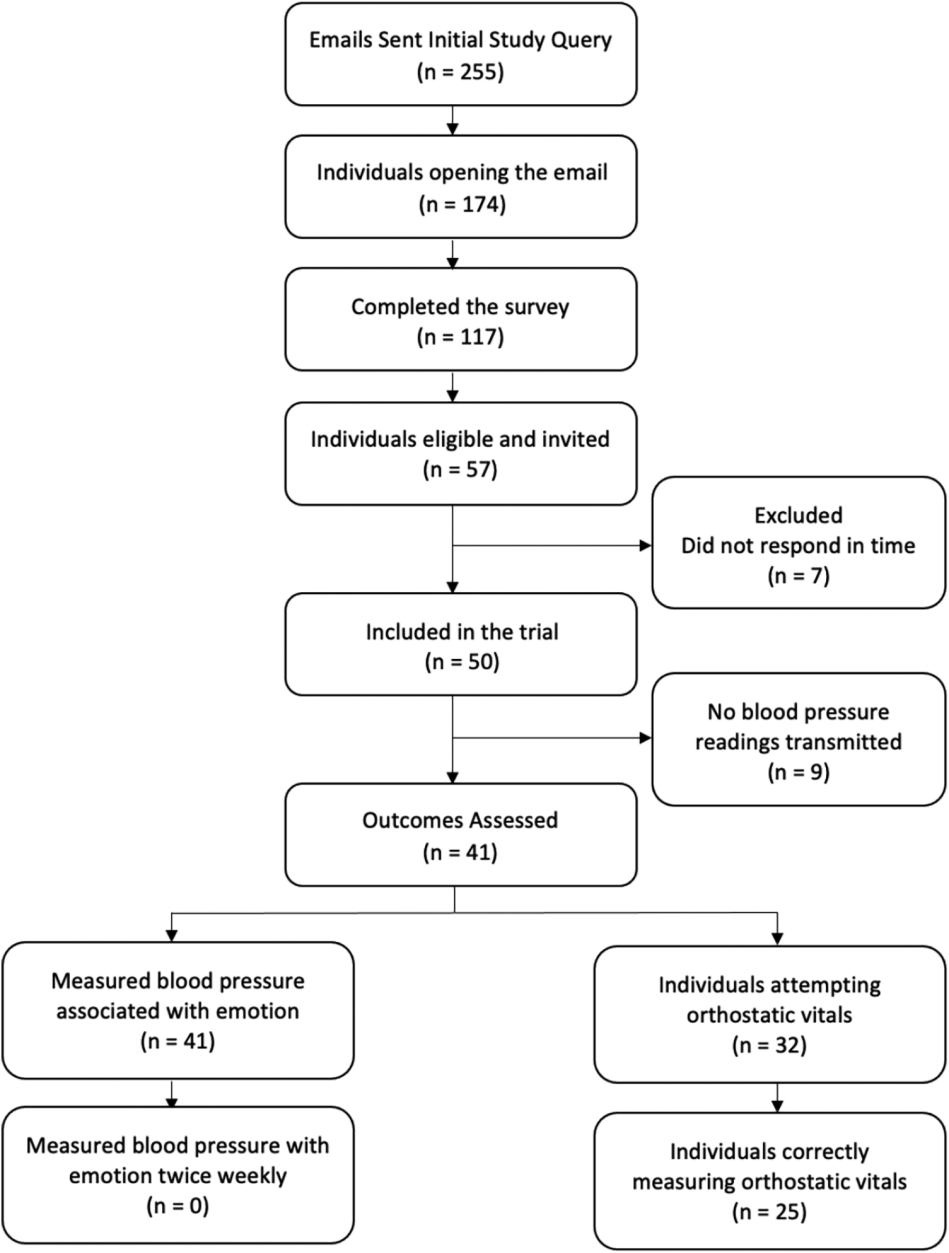
Selection process and usability of the study.

From a broad network of academic and community-based partners, 255 people were contacted by email for participation in the study. 174 individuals opened the email, with 117 individuals completing the eligibility survey. 57 individuals were invited to the next step and 50 participants were selected based on a balance of age and gender for the study groups (Figure 1). These individuals provided digital informed consent online and were subsequently sent the study device via mail post and study smartphone application for virtual enrollment. The average age of the 50 participants was 44.8 (SD 11.1) years, 50% were female, and 16% were on antihypertensive medications. Full baseline demographics of the study participants are in Table 1.

**Table 1:** Baseline characteristics of study participants.

Characteristics	Participants (*n* = 50)
Age, mean (SD)	44.8 (11.1)
Female sex, %	50%
Race	
Asian, *n* (%)	8 (16%)
Black/African American, *n* (%)	1 (2%)
White/ Caucasian, *n* (%)	35 (70%)
Other, *n* (%)	5 (10%)
Not provided, *n* (%)	1 (2%)
Prior diagnosis of hypertension, *n* (%)	14 (28%)
On hypertension medications, *n* (%)	8 (16%)
History of type 2 diabetes, *n* (%)	1 (2%)
History of congestive heart failure, *n* (%)	0 (0%)
Physical activity level	
Sedentary (little or no exercise), *n* (%)	10 (20%)
Lightly active (light exercise 1–3 days), *n* (%)	17 (34%)
Moderately active (moderate exercise 3–5 days), *n* (%)	21 (42%)
Very active (hard exercise 6–7 days), *n* (%)	2 (4%)
Smoking status	
Never smoked, *n* (%)	37 (74%)
Former smoker, *n* (%)	11 (22%)
Current smoker, *n* (%)	2 (4%)
Alcohol consumption	
Never, *n* (%)	8 (16%)
Monthly or less, *n* (%)	10 (20%)
2 to 4 times a month, *n* (%)	10 (20%)
2 to 3 times a week, *n* (%)	14 (28%)
4 or more times a week, *n* (%)	8 (16%)
Highest level of education	
High School/GED, *n* (%)	1 (2%)
Some College, *n* (%)	12 (24%)
Bachelor's Degree, *n* (%)	14 (28%)
Master's Degree, *n* (%)	11 (22%)
Advance graduate work (Ph.D., MD, etc), *n* (%)	12 (24%)

### Usability

Of the 50 individuals included in the trial, 9 did not transmit BP readings during the study. The mean age of the 41 individuals who transmitted BP measurements was 42.2 (SD 9.8) years, and the mean age of the 9 individuals who did not transmit BP measurements was 56.8 (SD 8.6) years. The difference in age was statistically significant (*p* < 0.05). The results of the pre—study survey demonstrated a non-significant difference between the group that transmitted data and the group that did not transmit data as 19 out of the 41 individuals who transmitted data owned other mobile health sensors/devices or activity monitors and 4 out of the 9 individuals who did not transmit data owned other mobile health sensors/devices or activity monitors. The 41 individuals who transmitted data were included in the downstream analysis. The median number of days that each participant transmitted BP readings was 11, with an interquartile range (IQR) of 8.5–17.5. The median number of total BP measurements per individual taken during the trial was 37, with an IQR of 24–49.5. Regarding total measurements per day, Monday and Wednesday had the most measurements with 263 and 245, respectively, while Saturday and Sunday had the fewest measurements with 148 and 151, respectively (Supplementary Figure S1). Out of 41 participants that transmitted data, 35 participants (85%) answered the post- study survey. 29 out of 35 participants (83%) answered “Yes” to the question “Did you learn anything about your blood pressure during this study?” When the same group was asked what the most difficult aspect of this study was, 11 out of 35 mentioned the size or appearance of the device, 4 out of 35 mentioned difficulties with the study instructions and 7 out of 35 mentioned difficulties remembering to take BP measurements.

### Daily BP variation

**Figure 2:**
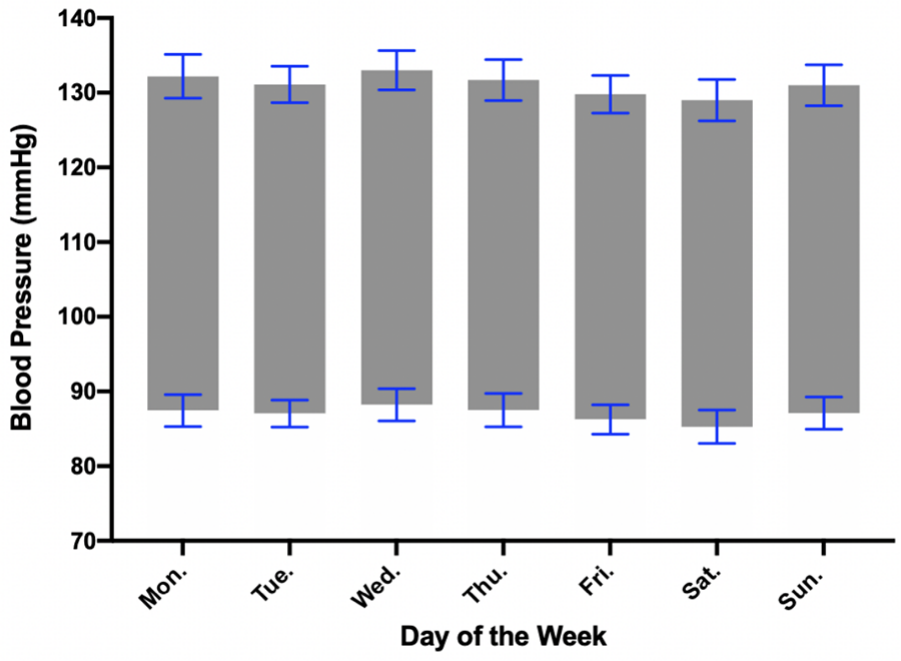
Blood pressure variation with day of the week. Average daily blood pressure was calculated from the 37 individuals who transmitted at least 10 total measurements. Wednesday was associated with the highest average systolic and diastolic BP while Saturday was associated with the lowest average systolic and diastolic BP. Error bars represent standard error of the mean (SEM).

Average systolic and diastolic BP variation with day of the week is shown in Figure 2. Only the 37 participants who transmitted a minimum of 10 BP recordings were included in the calculation. Single BP measurements were performed at each recording, and BP measurements were not performed in duplicate or triplicate. BP measurements associated with emotion and orthostatic measurements were excluded. No significant variation was seen when comparing average daily systolic and diastolic measurements. Wednesday was associated with the highest average systolic BP at 133.0 (SEM 2.6) mmHg and Saturday was associated with the lowest average systolic BP at 129.0 (SEM 2.8) mmHg. Wednesday was associated with the highest average diastolic BP at 88.2 (SEM 2.1) mmHg, and Saturday was associated with the lowest average diastolic BP at 85.3 (SEM 2.2) mmHg.

### Orthostatic vital signs

**Figure 3:**
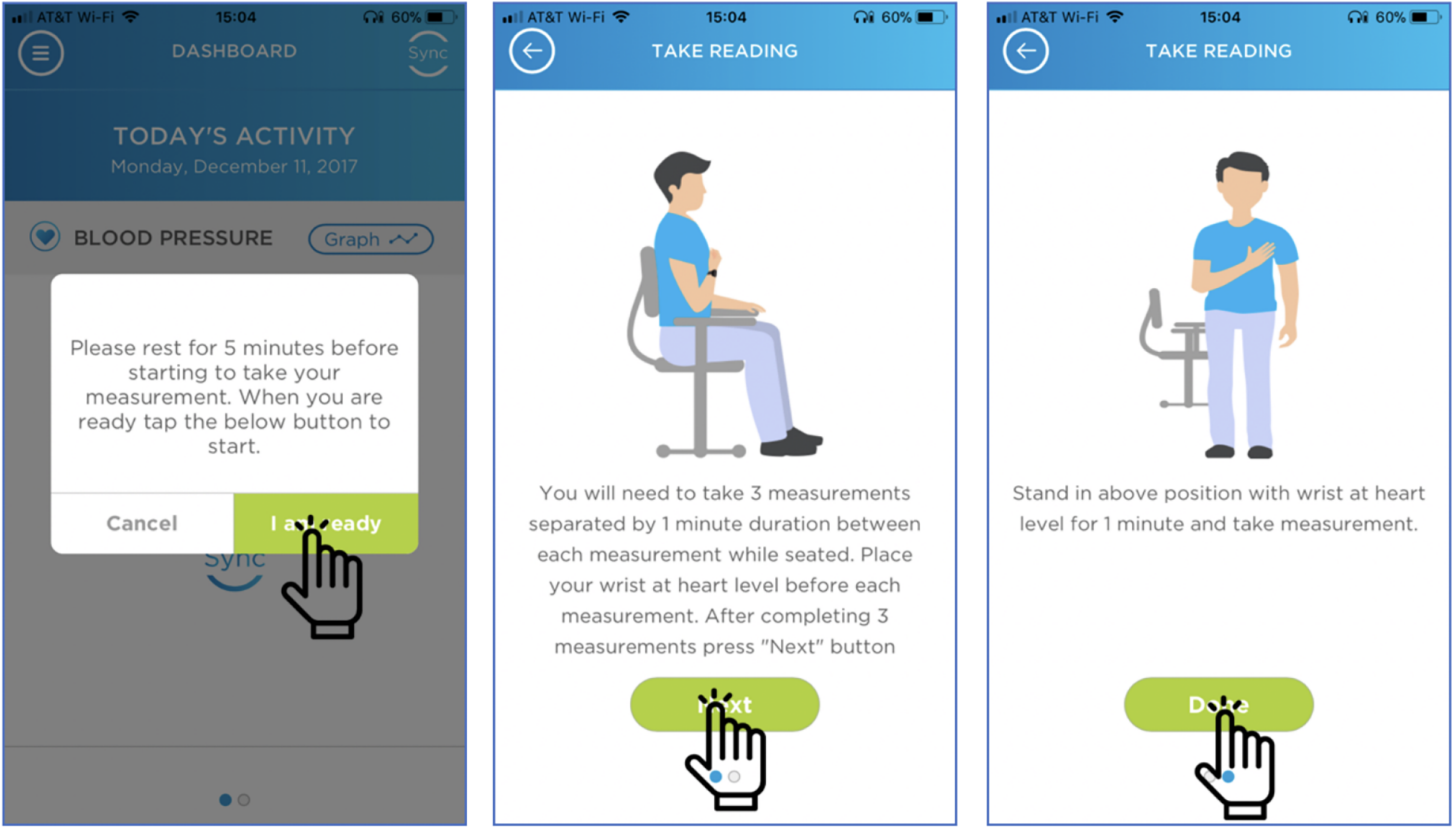
Digital instructions to perform orthostatic vital signs. These are the visual cues that participants were asked to follow when monitoring orthostatic vital signs. With the aid of visual cues, 25 out of 32 participants were able to perform orthostatic vital signs correctly.

Participants were asked to measure orthostatic vital signs at least twice during the 4-week study via digital cues provided on their smartphone application as seen in Figure 3. The correct measurement of orthostatic vital signs was determined based on a consensus statement on the definition of orthostatic hypotension (17). A positive result was defined as a drop in systolic BP of ≥20 mmHg or diastolic BP of ≥10 mmHg with standing as compared to the average of the three sitting BP measurements. Out of the 41 participants who transmitted data, 32 participants attempted orthostatic vital signs, with 25 participants correctly measuring orthostatic vital signs at least once during the study. Among those 25 participants, the average number of correctly measured orthostatic vital signs was 2.4, and the median number of correctly measured orthostatic vital signs was 1 (IQR of 1–3). Eleven participants followed study guidelines and correctly measured orthostatic vital signs at least twice during the study. We did not have participants document the presence or absence of symptoms in the smartphone application when recording orthostatic BP.

### BP variation with extremes of emotion

**Figure 4:**
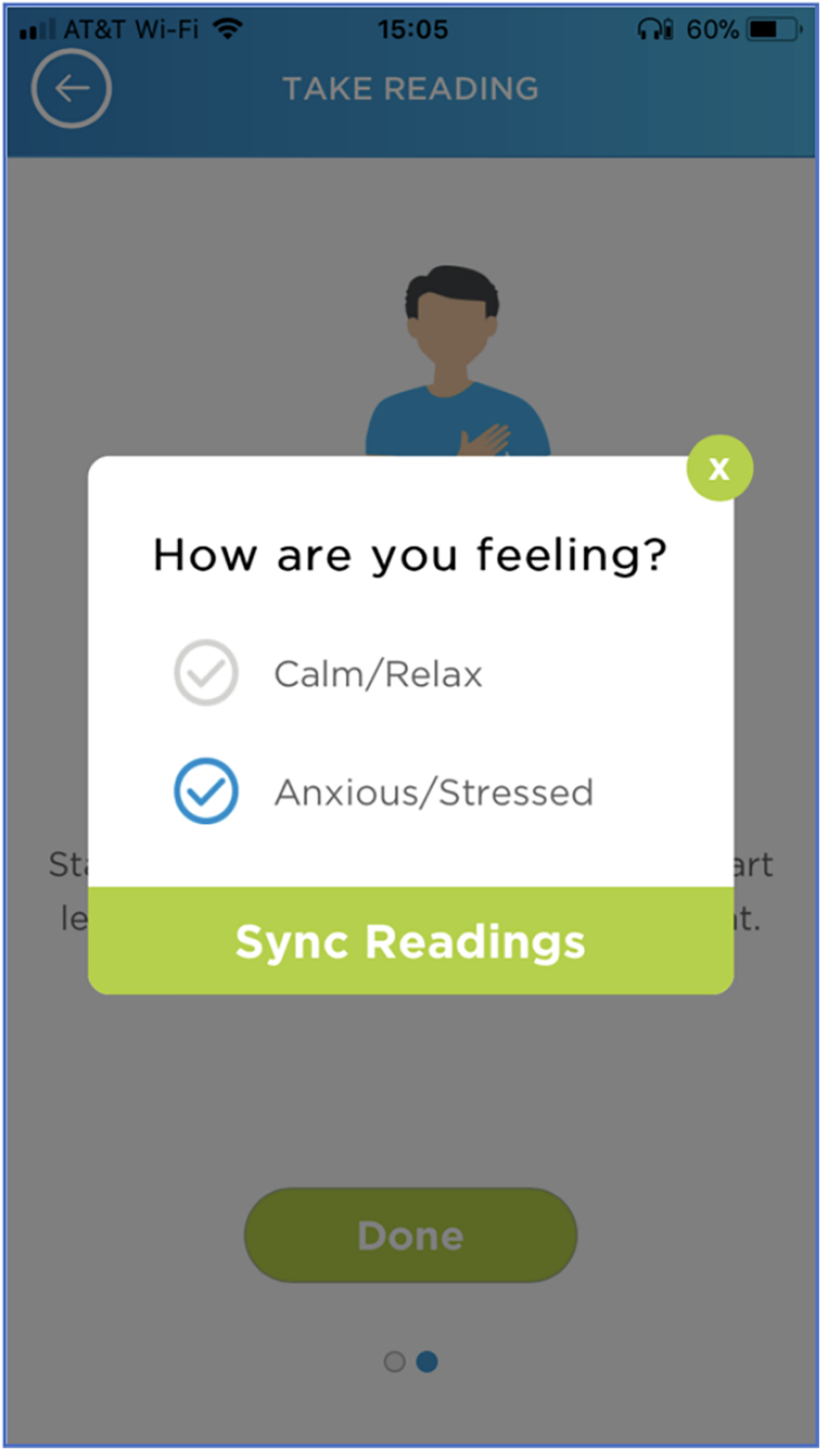
Digital cues on smartphone application to record BP associated with emotion. The visual interface that participants used on their smartphone when recording BP associated with an emotional state, either calm or stressed.

**Figure 5:**
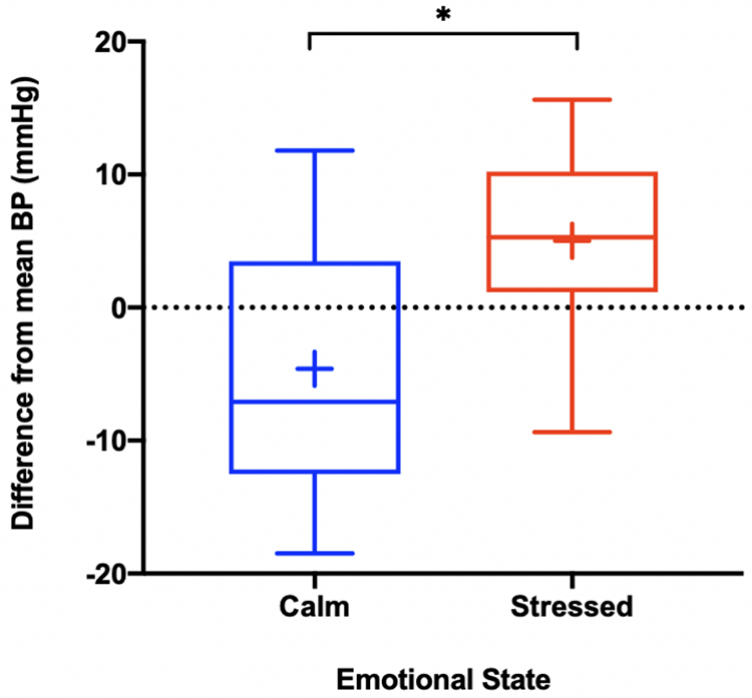
Bp associated with emotion. * = *p* < 0.005. The above box and whisker plots represent the median, interquartile range and standard deviation of the difference between participants average daily BP and BP associated with emotion, with the blue plot representing calm and the red plot representing stressed. The blue plus sign (+) represents the mean difference between participants average daily BP and BP associated with calm (−4.6 mmHg), while the red plus sign (+) represents the mean difference between participants average daily BP and BP associated with stressed (+5.0 mmHg). The associated mean differences were statistically significant as indicated above.

Participants were asked to record their BP with an emotional state, calm or stressed, at least twice per week with the aid of visual cues on the smartphone application Figure 4. 24 participants transmitted BP readings associated with emotion. 20 participants transmitted at least one BP reading associated with a calm emotional state, and 12 participants transmitted at least one BP reading associated with a stressed emotional state. No participants measured their BP twice weekly with emotion as detailed in the study guidelines. For all participants who measured their BP associated with emotion, their average daily BP was calculated and subtracted from their average BP associated with either calm or stressed emotional state and graphed. The median difference between participants' BP associated with calm and average daily BP was −7.1 (IQR −12.5–3.4) mmHg, while the median difference between participants' BP associated with stressed and average daily BP was +5.3 (IQR 1.2–10.2) mmHg. For completeness we also reported the mean difference between participants' BP associated with calm and average daily BP which was −4.6 mmHg, while the mean difference between participants' BP associated with stressed and average daily BP and +5.0 mmHg. The mean differences between calm and stressed emotional states were statistically different (*p* < 0.005) Figure 5.

### BP variation with time of day

**Figure 6:**
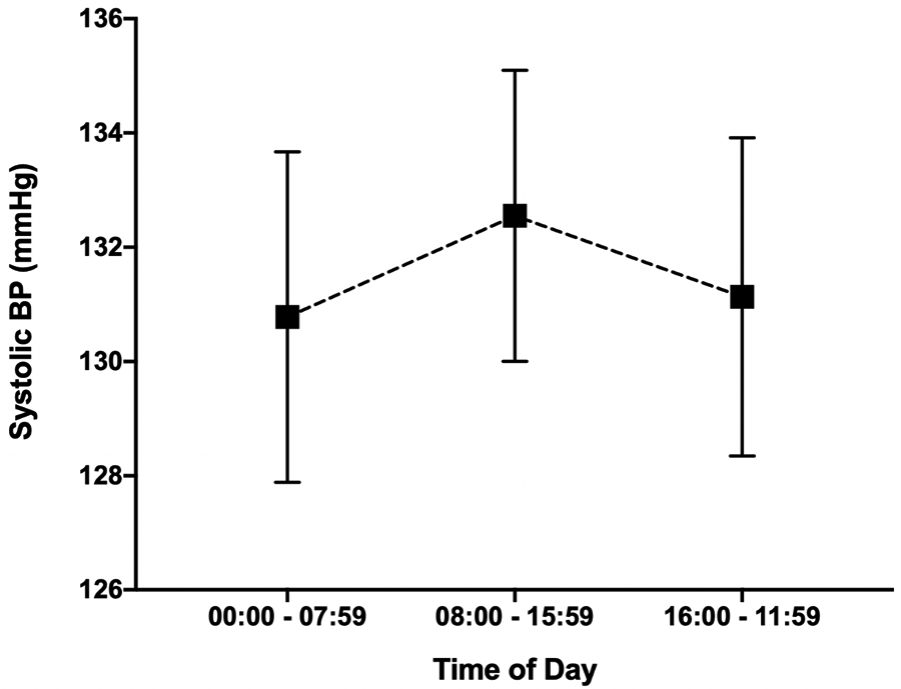
Blood pressure variation with time of day. The variation in average systolic blood pressure from midnight to 8 AM, 8 AM to 4 PM and 4 PM to midnight is graphed above. BP associated with each segment of the day was not statistically significant (*p* = 0.89).

37 participants transmitted at least 10 measurements over the course of the 4-week study. Data from those 37 participants was used to analyze systolic blood pressure variation with time of day. 12 out of 37 participants failed to transmit a BP reading from midnight to 8 AM. For these participants, their average blood pressure over the course of the study was substituted. All 37 participants transmitted a BP reading for segments 8 AM to 4 PM and 4 PM to midnight. The mean systolic BP for each segment of the day is graphed in Figure 6. The segment from 8AM to 4 PM was associated with the highest average systolic BP at 132.6 mmHg (SEM 2.5) and the segment from midnight to 8 AM was associated with the lowest average systolic BP at 130.8 mmHg (SEM 2.9). The BP variation associated with each segment of the day was not statistically significant (*p* = 0.89).

## Discussion

Out-of-office blood pressure monitoring has been shown to predict long term cardiovascular outcomes better than in-clinic BP monitoring and its use is supported in the most recent guidelines (18). However, these devices are only beneficial if participants use them, and thus highlights the importance of studies evaluating the usability of ambulatory BP devices. Our findings are consistent with prior studies demonstrating that wearable BP devices can be used for an extended period of time (19). The average age of the group that transmitted data was lower than the age of the group that did not transmit data and highlights the importance of appropriate patient selection for out-of-office blood pressure monitoring and helps identify patients who might need additional instruction.

### Out-of-office orthostatic BP monitoring via in-app instruction

Our study adds to prior research by providing a foundation to evaluate the usability of in-app instructions for out-of-office orthostatic BP monitoring. Although orthostatic BP is typically measured in the clinical setting, orthostatic BP measured outside of the clinical setting has been shown to be feasible and increase detection rates for orthostatic hypotension compared to traditional in-office testing (11, 13). However, this is the first study of its kind to provide instructions for measurement of orthostatic BP solely via a smartphone application and without in-person instruction. While we cannot verify that participants were positioned correctly during the individual measurements (sitting and standing), the smartphone application provided both written and visual cues to aid with correct positioning. Notably, the visual cues on the smartphone application differ slightly when compared to the user manual as the smartphone application does not show the arm with the wrist cuff being supported by the contralateral arm. Over half of the participants that attempted orthostatic vital signs timed the BP measurements appropriately, which was defined as less than or equal to 3 min between each of the 4 consecutive measurements. This finding cannot be understated as orthostatic hypotension can often go unrecognized or incorrectly diagnosed and is associated with increased morbidity and mortality (20).

### BP associated with calm and stressed emotional states

Our study was able to further expand on the effects of stress and calm on out-of-office BP. By syncing the wrist BP monitor with a smartphone application, participants were able to select their emotional state prior to taking a BP measurement. When compared to each participants average BP over the study, their associated calm BP readings were on average approximately 5 mmHg lower and their stressed BP readings were approximately 5 mmHg higher. These findings are consistent with a prior study by Tomitani et al. which showed a difference of 9.3 ± 2.1 mmHg on systolic BP measurement between positive and negative emotional states using WBPM devices (15). Since our study was decentralized and without in-person instruction, logging one's emotion state was streamlined and done completely within the smartphone application. Although participants did not strictly follow study guidelines regarding the frequency of obtaining BP associated with emotion, the majority were able take at least one BP measurement associated with emotion.

### BP trends with time of day and day of the week

While we were able to appreciate trends in BP associated with time of day and day of the week, the variation did not reach statistical significance. We acknowledge that using the individual average BP rather than leaving that day blank, in the circumstance when a participant did not have a BP measurement for that specific day, may potentially bring to an underestimation of the true daily BP variation. The general trend for low BP on Saturday when compared to other days of the week was observed, underscoring the role of work-related stress on out-of-office BP. Our study showed a trend toward decreased BP at night however did not reach statistical significance potentially due to decreased BP transmissions overnight as the act of recording BP with WBPM devices is an active process. While we would have liked to have a cuff that measured BP automatically every hour, especially during overnight hours, this was not an available feature for the chosen oscillometric cuff. This highlights the importance of future studies evaluating wearable passive BP monitors that can transmit data while sleeping.

### Usability

After reviewing a post study survey regarding the usability of the device, the main limitations were the size and appearance of the wrist monitor. While smaller than the traditional arm cuff, the wrist cuff was still larger than most wrist watches. As a result of its size, participants mentioned that it was uncomfortable to wear daily. While not mentioned in the survey, it is also plausible that since the device only comes in one color and looks like a medical device, participants might have felt stigmatized while wearing it. Another barrier to transmitting BP readings mentioned in the survey was remembering to take the measurements. Several participants even recommended incorporating cues into the smartphone application to trigger BP measurements which could be an area for further study. While our study was decentralized and directions for participating in the study were provided virtually rather than in person, difficultly following the study instructions was only mentioned in 4 out of 35 post survey responses.

### Limitations

As this was primarily a usability study, one of the major limitations to the study is that we collected very little data from the 9 participants who did not transmit data during the study. Only 2 of the 9 participants responded to the post study survey, with one citing difficulty connecting the phone to the device as the most difficult aspect of the study while the other participant mentioned difficultly adjusting the band as the most difficult part of the study.

## Conclusions

This prospective digital study shows that complex BP measurements, such as orthostatic vital signs and BP associated with emotion, can be performed outside of the clinic setting using digital instructions provided via a smartphone application synced with wearable BP technology. As BP monitors get smaller and easier to use, future studies can evaluate the utility of continuous, passive BP monitors especially when monitoring BP while sleeping. Since no participants recorded BP associated with emotion twice per week as written in the study guidelines, additional research should be done to evaluate the role of in-app cues and reminders to record out-of-office BP.

## Data Availability

The raw data supporting the conclusions of this article will be made available by the authors, without undue reservation.

## Supplementary material

Supplementary Figure S1 Total BP Measurements per Day. Total number of BP measurements per day throughout the course of the study excluding orthostatic BP measurements and BP associated with emotion.
