# Supplementary material for: A pilot study exploring novel contexts for out-of-office blood pressure measurement
Source: Front Cardiovasc Med. 2024 Feb 23;11:1351746. doi: 10.3389/fcvm.2024.1351746 (PMC10920340; doi:10.3389/fcvm.2024.1351746)
Supplement: Supplementary Figure S1 — Total BP Measurements per Day. Total number of BP measurements per day throughout the course of the study excluding orthostatic BP measurements and BP associated with emotion. [file Datasheet1.pdf]

SUPPLEMENTAL INFORMATION

Supplemental Information: Post-Study Questionnaire

*Objectives: ease of using **device**, ease of using **module**, discoveries about **blood pressure***

1. Were you able to first set up the BP device with ease?
   If not, what was the most significant issues?

2. Did you need to call for support to set up device or help syncing device?
   If yes, were your questions answered adequately?

3. Was the BP App easy to navigate?
   If not, where did you encounter difficulties:

4. In terms of the BP activity requirements (asks), where they too many, just right, or too few.

5. On finishing the pilot program, did you discover anything new about your BP?

6. Did you plan to discuss the findings with your health care provider?

Supplemental Figure 1: Total BP Measurements per Day

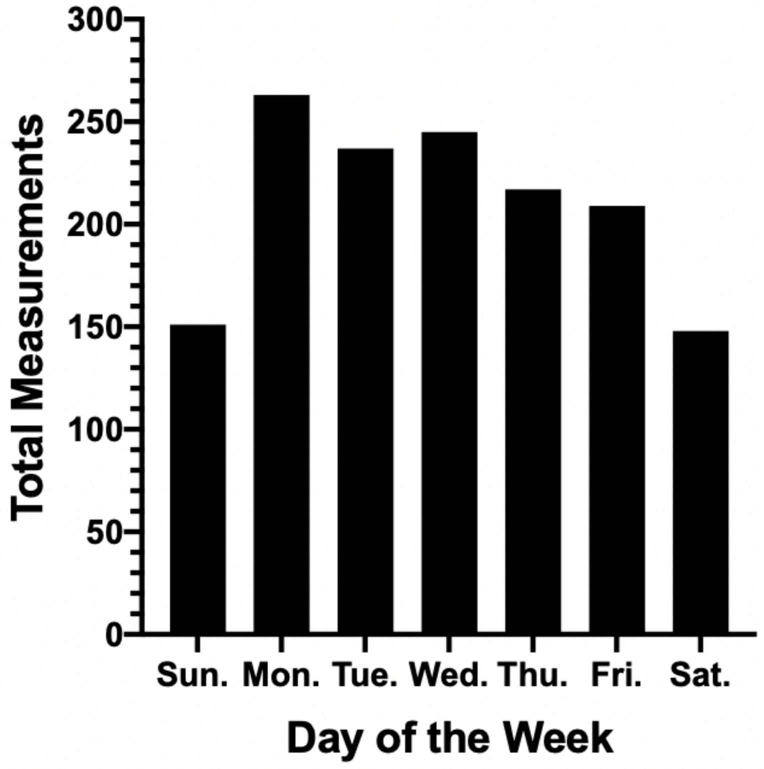

Total number of BP measurements per day throughout the course of the study excluding orthostatic BP measurements and BP associated with emotion.
